# Epstein-Barr Virus and Human Papillomavirus Coinfection in Colorectal Carcinoma: Systematic Review and Meta-Analysis of the Prevalence

**DOI:** 10.3390/microorganisms12112117

**Published:** 2024-10-23

**Authors:** Ana Banko, Ivana Lazarevic, Danijela Miljanovic, Maja Cupic, Andja Cirkovic

**Affiliations:** 1 Institute of Microbiology and Immunology, Faculty of Medicine, University of Belgrade, 11000 Belgrade, Serbia; ivana.lazarevic@med.bg.ac.rs (I.L.); danijela.karalic@med.bg.ac.rs (D.M.); maja.cupic@med.bg.ac.rs (M.C.); 2 Institute for Medical Statistics and Informatics, Faculty of Medicine, University of Belgrade, 11000 Belgrade, Serbia; andja.cirkovic@med.bg.ac.rs

**Keywords:** colorectal carcinoma, coinfection, Epstein–Barr virus (EBV), human papillomavirus (HPV), prevalence, copresence, oncoviruses

## Abstract

Colorectal cancer (CRC) is one of the most common types of cancer worldwide. In addition to known risk factors, oncoviruses have attracted exceptional attention from recent research. Numerous hypotheses on interactions between the Epstein–Barr virus (EBV) and Human papillomavirus (HPV) in CRC are still based on sparse prevalence data of these coinfections. The aim of this study was to perform a comprehensive literature search regarding EBV/HPV coinfection in patients with CRC and to provide its prevalence in the target population. Three electronic databases (PubMed, SCOPUS, and WoS) were searched, and after a double reviewer check, six publications were included in the qualitative and quantitative analyses. This systematic review showed the limited number of studies dealing with the prevalence and role of EBV/HPV coinfection in CRC and the heterogeneity of methodology and reported results. However, in a total of 635 patients, it also showed that the identified 15% EBV/HPV prevalence in CRC (15%, 95% CI: 7–23%) could suggest that further investigations are needed. Histopathologically, all coinfected CRCs were adenocarcinomas, from intermediate to intermediate and high grade, reported across four studies. Increased knowledge about the infectious origin of various cancers, including CRC, has the potential to enhance the vigilance of scientists to design future large-scale multicenter prospective studies.

## 1. Introduction

Colorectal cancer (CRC) is one of the most common types of cancer worldwide. With its high prevalence and very slow progression, it is responsible for more than 900,000 deaths per year [1,2]. Every person has an approximately 4% lifetime risk of developing CRC [3]. In addition to known risk factors, including age, family history of CRC, nutritional lifestyle, personal history of inflammatory bowel disease, obesity, smoking, and alcohol consumption, infectious agents such as oncoviruses attract significant attention from recent research [1,4,5].

There is an increasing number of reports suggesting associations between viral infections and human malignancies with an estimated 20% of cancers attributed to viral infections [6,7]. When it comes to CRC, the Epstein–Barr virus (EBV), human papillomavirus (HPV), and John Cunningham virus (JCV) were the most reported oncoviruses in previous studies, but with very heterogeneous results. Additionally, a wide range of relative risk estimations between specific viral infections and CRC development demonstrates how difficult is to prove a causal relationship between infection and the risk of developing CRC [1,5]. A clear definition of viral etiology will therefore be known only after currently lacking large-scale, methodologically rigorous time-trend epidemiological studies.

Modern understanding of different human oncogenic DNA viruses highlights several common biological features: establishing latent infection, dependency on the infected cell replication capacities, capability to disrupt the cell cycle, interfering with epithelial-mesenchymal transition (EMT)-/epidermal growth factor receptor (EGFR)-associated pathways, and influencing the Wnt/β-catenin pathway [1]. EBV infection influences some cell signaling pathways which could lead to modification of the host genome‘s methylation and the overexpression of genes that are involved in cytokine regulation, cytoskeleton formation, cell proliferation and adhesion [1,8]. Additionally, activated epigenetic mechanisms drive the elevation of transcription factor levels including the T-cell factor (TCF) and the lymphoid enhancer factor (LEF1). As this epigenetic mechanism remains active even after viral load resolution, some authors have suggested that the EBV has the potential to induce “hit-and-run” mutagenesis in a cell and contribute to CRC oncogenesis or even metastasis [1]. Further insights into the role of exogenous microRNAs in CRC development have also been described. As it was previously suggested that a cluster of 22 EBV microRNAs, called miR-BamHI-A rightward transcripts (miR-BARTs), could promote cancer development, Meng et al. [9] identified EBV-miR-BART18-3p as highly expressed in CRC and closely associated with the pathological and advanced clinical stages of CRC. The mechanism behind this oncogenesis is based on the lipogenesis pathway. When it comes to HPV-induced oncogenesis, the role of HPV in CRC is also not clear enough. Mutations in p53 represent a CRC characteristic. Although it seems that CRC tissues infected with HPV usually have an intact Tp53, the p53 functions are disrupted which leads to the hypothesis that HPV still inactivates p53, thus promoting cancer [1,10]. Furthermore, evidence suggests a relationship between HPV infection and pro-oncogene modulation of the Wnt/β-catenin pathway via E6 and E7 viral oncoproteins [11]. In addition, it has been described that HPV can also change the biochemistry in the infected cell. E7 can modify M2 pyruvate kinase, promoting glycolysis and reducing the need for oxygen in the infected cell [1]. This is of particular interest because it could ensure cancer cell survival in hypoxic conditions especially diets rich in sugar, and metabolic conditions are associated with an increased risk of CRC [12]. In addition to the described ideas, there are also other possible transformation mechanisms like chromosomal instability induced by E7, overexpression of the catalytic subunit of telomerase (TERT) and many more that have to be further investigated in the context of CRC evolution.

While the exact role of individual viruses in CRC pathogenesis is pending, the role of interactions between EBV and high-risk HPV types in different carcinomas is also of particular interest [13,14,15,16]. There is a hypothesis that when these two viruses act together in infected epithelial cells, they could accelerate each other’s oncogenic potential [6,17,18]. For example, EBV lytic reactivation could be stimulated by HPV-induced elevation of KLF4 expression [19]. On the other hand, EBV has the capacity to reprogram lethally differentiating cells to support cell cycle progression by HPV oncogenes [20]. The EBV could also enhance invasiveness of the epithelial cells that express E6 and E7 HPV oncoproteins, thus enabling the interaction of both EBV and HPV oncogenes [21]. Finally, it has been suggested that EBV infection is associated with an increased risk of HPV DNA integration into the host genome which is one of the steps in HPV-induced cell transformation [22].

Despite numerous hypotheses of biological mechanisms of interaction between EBV and HPV, data about the exact prevalence of these coinfections in CRC, for example as to which virus is responsible for the initial infection and which one is responsible for “superinfection”, and the association between the cancer phenotypes and dual infections are missing. The aim of this study was therefore to perform a comprehensive literature search regarding EBV/HPV coinfection in patients with colorectal cancer and to provide its prevalence in the target population.

## 2. Materials and Methods

### 2.1. Study Design

This study, which was previously registered at PROSPERO with the registration number CRD42024581495, was performed according to the recommended PRISMA protocol for systematic reviews and meta-analyses [23].

### 2.2. Eligibility Criteria

Studies that evaluated EBV and HPV coinfection in patients who suffered from colorectal carcinoma were included in this systematic review. Studies were excluded if they: (1) examined populations other than human (animals, cell lines), (2) did not evaluate EBV and HPV coinfection, and (3) were not original articles (narrative reviews, systematic reviews, meta-analyses, editorials, comments, correspondences, books, chapter in a book, abstracts, etc.).

Two researchers with expertise in conducting systematic reviews and meta-analyses (AB, AC) developed and ran the search in three electronic databases: PubMed, Web of Science (WoS), and SCOPUS until 15th July 2024. The following keywords were used for the search query in PubMed: (Colorectal Cancer or Colorectal Carcinoma or Colorectal Tumor* or Neoplasms, Colorectal or Caecal carcinoma* or Rectal carcinoma* or Caecal neoplasm* or Rectal neoplasm* or Rectal cancer or Caecal cancer or Colon cancer* or Colon carcinoma* or Colon neoplasm* or Bowel cancer* or Bowel carcinoma* or Bowel neoplasm* or CRC) and (Epstein Barr Virus or Epstein-Barr Virus or EBV or Burkitt’s Lymphoma Virus or Herpesvirus 4, human or HHV4) and (HPV Human Papillomavirus or HPV, Human Papillomavirus Viruses or Human Papilloma Virus or Human Papillomavirus or HPV). In SCOPUS: (TITLE-ABS-KEY (“Colorectal Cancer”) OR TITLE-ABS-KEY (“Colorectal Carcinoma”) OR TITLE-ABS-KEY (“Colorectal Tumor”) OR TITLE-ABS-KEY (“Neoplasms, Colorectal”) OR TITLE-ABS-KEY (“Caecal carcinoma”) OR TITLE-ABS-KEY (“Rectal carcinoma”) OR TITLE-ABS-KEY (“Caecal neoplasm”) OR TITLE-ABS-KEY (“Caecal cancer”) OR TITLE-ABS-KEY (“Colon cancer”) OR TITLE-ABS-KEY (“Colon carcinoma”) OR TITLE-ABS-KEY (“Colon neoplasm”) OR TITLE-ABS-KEY (“Bowel cancer”) OR TITLE-ABS-KEY (“Bowel carcinoma”) OR TITLE-ABS-KEY (“Bowel neoplasm”) OR TITLE-ABS-KEY (“CRC”)) AND (TITLE-ABS-KEY (“Epstein Barr Virus”) OR TITLE-ABS-KEY (“Epstein–Barr Virus”) OR TITLE-ABS-KEY (“EBV”) OR TITLE-ABS-KEY (“Burkitt’s Lymphoma Virus”) OR TITLE-ABS-KEY (“Human herpesvirus 4”) OR TITLE-ABS-KEY (“HHV4”)) AND (TITLE-ABS-KEY (“HPV Human Papillomavirus”) OR TITLE-ABS-KEY (“HPV, Human Papillomavirus”) OR TITLE-ABS-KEY (“Human Papilloma Virus”) OR TITLE-ABS-KEY (“Human Papillomavirus”) OR TITLE-ABS-KEY (“HPV”)). And, in WoS: (ALL = (“Colorectal Cancer”) OR ALL = (“Colorectal Carcinoma”) OR ALL = (“Colorectal Tumor”) OR ALL = (“Neoplasms, Colorectal”) OR ALL = (“Caecal carcinoma”) OR ALL = (“Rectal carcinoma”) OR ALL = (“Caecal neoplasm”) OR ALL = (“Caecal cancer”) OR ALL = (“Colon cancer”) OR ALL = (“Colon carcinoma”) OR ALL = (“Colon neoplasm”) OR ALL = (“Bowel cancer”) OR ALL = (“Bowel carcinoma”) OR ALL = (“Bowel neoplasm”) OR ALL = (“CRC”)) AND (ALL = (“Epstein Barr Virus”) OR ALL = (“Epstein–Barr Virus”) OR ALL = (“EBV”) OR ALL = (“Burkitt’s Lymphoma Virus”) OR ALL = (“Human herpesvirus 4”) OR ALL = (“HHV4”)) AND (ALL = (“HPV Human Papillomavirus”) OR ALL = (“HPV, Human Papillomavirus”) OR ALL = (“Human Papilloma Virus”) OR ALL = (“Human Papillomavirus”) OR ALL = (“HPV”)). Publications in English were only considered. In addition, reference lists of the articles identified through electronic retrieval and relevant reviews and editorials were manually searched to check for more potentially relevant articles.

### 2.3. Article Screening and Selection

Publications were screened for inclusion in the systematic review independently by two reviewers (DM, IL), first through title and abstract, and afterward through full-text reading. All disagreements were resolved by discussion at each stage with the inclusion of a third reviewer (AB). Studies were included in the full-text screening step if either reviewer identified the study as potentially eligible or if the abstract and title did not have sufficient information for exclusion.

### 2.4. Data Abstraction and Quality Assessment

Two reviewers (AB, AC) independently abstracted the following data: author(s), year of publication, country of research, study design, population characteristics (size, age, gender, lymph node involvement, and distant metastasis), number of patients with EBV and HPV coinfection and their characteristics (histological type, grade, stage, and localization of colorectal cancer), and tissue type and detection method for EBV and HPV, as well as HPV genotype in coinfections. Independent reviewers used previously designed protocols to select and abstract the data. Authors of relevant articles were contacted to obtain unavailable manuscripts and/or missing data. The Newcastle–Ottawa tool (NOS) [24] for observational studies was used. The NOS tool considers selection, comparability, and exposure domains for case-control or selection, comparability, and outcome domains for cohort studies. The selection domain refers to the definition, selection, and representativeness of cases and controls in case-control studies, while it refers to the selection and representativeness of the exposed cohort, as well as to the proof of non-existence of the outcome at the beginning of the research, in cohort studies. The comparability domain refers to the way of controlling confounding variables for both study designs. The exposure domain refers to the source of data and the response rate. The outcome domain refers to the way of detecting the outcome, the duration of follow-up and the percentage of loss to follow-up. The study quality, according to NOS, was good (3 or 4 stars in selection AND 1 or 2 stars in comparability AND 2 or 3 stars in outcome/exposure domain, or ≥7 stars in total), fair (2 stars in selection AND 1 or 2 stars in comparability AND 2 or 3 stars in outcome/exposure domain, or 5–6 stars in total), or poor (0 or 1 star in selection OR 0 stars in comparability OR 0 or 1 star in outcome/exposure, or ≤4 stars in total).

### 2.5. Statistical Analysis

The primary outcome of this meta-analysis was to assess the prevalence of EBV and HPV coinfection in patients who suffered from colorectal cancer. The meta-analysis of the prevalence was performed using the inverse variance method. Data entered for each of the studies were the original prevalence from the included study and the standard error of the prevalence according to the equation √((p*(1 − p))/n), where p is the prevalence and n is the total number of patients in the study. Funnel plots assessed publication bias for each outcome (Supplementary Materials Figure S1: Funnel plot). A *p*-value ≤ 0.05 was considered to be statistically significant. Analyses were performed using Review Manager 5.3.

## 3. Results

**Figure 1:**
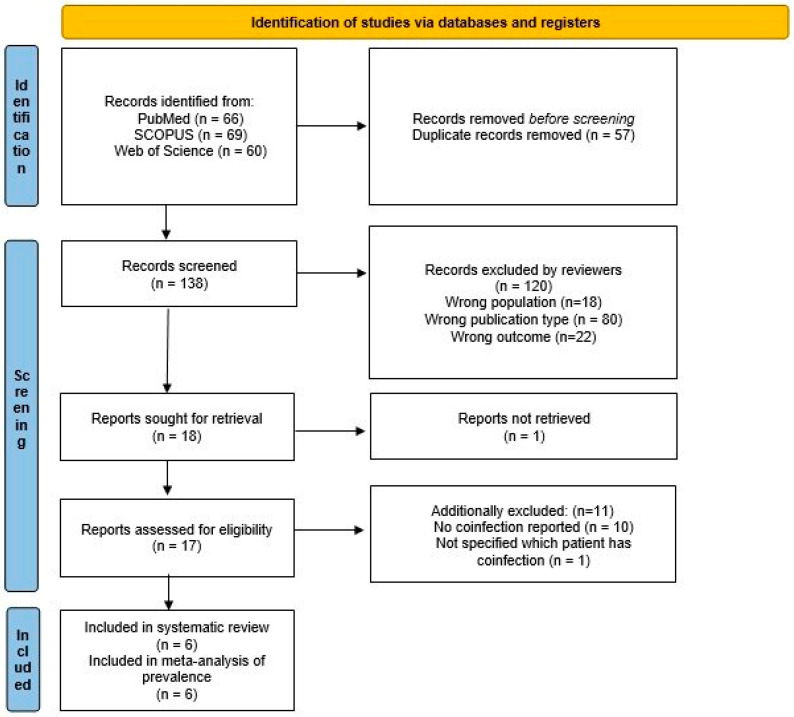
Flow chart presenting the selection process.

A total of 195 potentially eligible articles were found. Title and abstracts were evaluated for 138 articles, after duplicates (57) were removed. A total of 18 were retrieved, 17 were considered in full, after 120 articles were excluded according to previously defined exclusion criteria, and one was unavailable. Finally, 6 articles were selected for inclusion in the systematic review and meta-analysis of the prevalence. A flow diagram illustrating the selection process is presented in Figure 1.

Quality assessment of the included articles performed using the adapted version of the Newcastle–Ottawa scale is shown in Table 1. We have two studies with good and four with poor quality.

Characteristics of the included publications within the systematic review are presented in Table 2. They were published during the last decade, between 2013 and 2023, with a total of 635 patients who suffered from colorectal carcinoma. The EBV/HPV co-presence was reported as follows during this period: 8% in 2013, 16% and 17% in 2020, 28% in 2021, 2% and 17% in 2023. Only one study reported the design (cross-sectional), while this was not reported in the other five. The country of study origin differed for all included publications: the United States of America, Bosnia and Herzegovina, Syria, Lebanon, Iran, and Qatar. The average age of CRC patients was 58.4 years. There were 248 male and 222 female patients who suffered from colorectal cancer. Age and gender were not reported in one study. There were 91 CRC patients with EBV and HPV coinfection. All of them had adenocarcinoma as a histopathological type of CRC. They were of intermediate grade in two studies, and intermediate and high grade in two studies, while grade was unclear in two and not reported in one study. The tumor stage was reported in just one study and there was 1 patient with a pT1, 6 patients with a pT2, and 10 patients with a pT3 stage. The most commonly reported localization of colorectal cancer was in the rectum (19 cases). Other reported localizations were as follows: colon (13 cases), rectosigmoid (13 cases), sigmoid (5 cases), ascending colon (2 cases), hepatic flexure (2 cases), descending colon (1 case), transverse colon (1 case), cecum (1 case), and other localizations (14 cases). Localization was not specified in two studies. EBV and HPV were detected in frozen tissue in two studies using next generation sequencing (NGS) in one and polymerase chain reactions (PCR) in another as a detection method. In addition, they were detected in formalin-fixed paraffin-embedded (FFPE) tissue samples in four studies using as a detection method PCR only or PCR, a tissue microarray (TMA), and immunohistochemistry (IHC) in two studies each. All studies detected high-risk HPV genotypes except one that did not report HPV genotype: HPV16 was detected in 3, HPV18 in 5, HPV31 in 3, HPV33 in 2, HPV35 in 4, HPV45 in 2, HPV51 in 2, HPV52 in 3, HPV56 in 1, HPV58 in 2, and HPV59 in 1 case.

Additionally, we evaluated the obtained results from the included studies regarding EBV, HPV, and EBV/HPV coinfection and their association with colorectal carcinomas. The authors of five out of six papers selected by this systematic review attempt to determine the association between EBV/HPV coinfection and clinicopathological characteristics of CRC [17,18,26,27,28]. The evaluated CRC traits were as follows: histological type and tumor grade by 6/6 studies, gender and age (current age, 1/5, and age at initial diagnosis, 4/5) by 5/6 studies, localization by 4/6 studies, lymph node involvement by 2/6 studies, and the history of polyp presence, distant metastases, tissue class (formalin-fixed paraffin-embedded or frozen tissue), and sampling method (biopsy or surgery) by one study each. Only two of the included studies managed to prove the higher presence of EBV/HPV coinfection in malignant than in healthy control tissue samples [17,28]. In addition, only one study reported the presence of the association of the mentioned coinfection with the grade of the tumor [17]. Moreover, only one study found the predictor of higher CRC stage to be in the copresence of EBV and more than two HPV subtypes [28].

**Figure 2:**
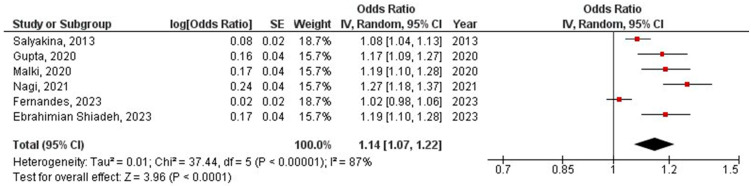
Meta-analysis of EBV/HPV coinfection prevalence in colorectal cancer population [17,18,25,26,27,28].

The obtained prevalence of EBV/HPV coinfection in the CRC population according to the six included studies was 15% with a 95% confidence interval from 7% to 23% (Figure 2). Sensitivity analysis was performed by excluding studies of good quality (OR = 1.16, 95% CI OR = 1.04–1.31, *p* = 0.010) (Supplementary Materials Figure S2: Sensitivity analysis with studies of poor quality), by excluding studies not performed in Asia (OR = 1.17, 95% CI OR = 1.04–1.31, *p* = 0.010) (Supplementary Materials Figure S3: Sensitivity analysis with studies performed in Asia), and by excluding studies not using FFPE or PCR (OR = 1.17, 95% CI OR = 1.06–1.29, *p* = 0.001) (Supplementary Materials Figure S4: Sensitivity analysis with studies using FFPE and PCR).

## 4. Discussion

The combined oncogenic effect of viral infections has been recognized as an oncogenic driver which has not been sufficiently explored so far. However, it is unequivocal that viruses are detected in an increasing number of cancers, including CRC. Moreover, studies have revealed or suggested a positive association between EBV or HPV infection and CRC [5,29,30]. To our knowledge, this is the first systematic review that summarizes results regarding EBV/HPV coinfection in CRC. Despite dozens of publications dealing with this topic, only a limited number of papers provided original data on coinfections. Facing this and several other limitations, it was still possible to provide the first meta-analysis of EBV/HPV coinfection prevalence in patients with CRC.

In a total of 635 patients, the overall prevalence of EBV/HPV coinfection was 15%. Ranging from 7–23%, the results were heterogeneous. Among the most important reasons were an insufficient number of subjects, a different selection of viral nucleic acid detection methods, the localization and grade of carcinoid tissue, and finally, a very narrow geographical focus. The majority of studies were on south-west Asian countries with the exception of USA and Bosnia and Herzegovina.

Although the role of coinfection is very intriguing, knowledge is mainly based on the theoretical aspects of the reviewed papers. So far, conclusions have been drawn from the data on the detection of individual viruses in CRC tissues. Thus, meta-analyses reported HPV prevalence between 14.1–60.8% with geographic variations and variations dependent on virus detection methods [31,32]. The highest prevalence was registered in South America and the lowest in Europe, while PCR was shown to be the most likely method for obtaining high prevalence values [31]. The EBV-positivity in the latest meta-analysis ranged from 0% in Africa to 30% in South America in CRC tissues [33]. Additionally, some authors reported even 46% of EBV findings in rectal cancer [34] and 52% in colon cancer [35]. The fact is, however, that the prevalence of EBV is lower than that of HPV when previous studies are considered. In addition to their retrospective nature, selection bias, different sample types, isolation and detection methods, various disease stages, and small sample sizes, there are additionally two important, but unresolved issues related to the testing of EBV presence. The first was observed using PCR assays, where possible contamination by EBV-positive inflammatory cells, but not cancer cells, was suggested [35,36]. The other is related to the dilemma of whether the presence of EBV in its latent, but not lytic stage in lymphoid aggregates within the tumor has an oncogenic significance. Therefore, different selections of target genes or proteins in the methodological protocols of different studies could lead to different interpretations of the results. In this systematic review, it was not possible to precisely determine the prevalence of EBV or HPV as mono-infections, mainly because the authors did not focus on the separation of mono and coinfections and thus did not report these results.

Previous studies that investigated HPV in CRC reported the presence of highly oncogenic HPV types including 16, 18, 31, 33, 35, 45, 51, 52, 56, and 58 [17,18]. A meta-analysis study based on the European population demonstrated HPV18 positivity in 47% of CRC cases [32]. Our systematic review did not reveal different results in relation to the identified HPV genotypes, with the dominance of HPV 18. This predominance together with HPV 16 is also in line with findings in other HPV-related cancers such as cervical cancer. However, the multiplex PCR assays that detect grouped high-risk HPV types that were used in most of the obtained studies make it impossible to accurately determine the prevalence of each HPV type individually.

To avoid biases associated with detection procedures, more state-of-the-art methods with higher sensitivity should be applied to detect viruses in CRC and other cancer tissues [37]. Thus, some authors have suggested a full NGS sequencing of CRC cells and analysis of any EBV nucleic acid fragments present [36]. In addition, some of the latest approaches in single-cell analysis could help in circumventing possible contamination by infiltrating immune cells [36]. Advances in massive parallel DNA sequencing and the latest bioinformatic protocols have already revealed potential CRC neoantigens in order to provide the basis for the development of immunotherapeutic strategies and cancer vaccines [38]. This emphasizes the importance of the implementation of new technologies in understanding the interactions of tumor cells with the immune system.

All of the mentioned study shortcomings are also applicable to those that reported EBV/HPV co-presence. In particular, there is a lack of focused identification of coinfections, so they were mentioned as secondary findings in a majority of the papers. Therefore, further analyses of the impact of that phenomenon were mostly missing and inconsistent. Salyakina et al. [25] found that EBV infection was common in pathologic T3 and in I or II stage CRCs, while HPV infection was common in CRC patients with a history of colon polyps and with cancer localized in the sigmoid, ascending colon and cecum. However, there was no association between EBV or HPV infection in colon and rectal carcinoma with gender, age at initial diagnosis, histological type, or ‘M’ and ‘N’ staging. Despite the fact that the same authors had found the presence of EBV/HPV coinfection, they did not evaluate the association of this coinfection with CRC presence or clinicopathological characteristics. Gupta et al. [18] detected EBV, HPV, and EBV/HPV coinfection in malignant tissue, but did not discover any relationship with CRC clinicopathological features (age, gender, tumor grade, tumor stage, or anatomical localization). EBV/HPV coinfection in intermediate and high-grade CRC samples was commonly detected in comparison with EBV + HPV-, EBV-HPV+, and EBV-HPV- by Malki et al. [17]. It is important that the same study did not detect either EBV or HPV in normal colorectal mucosa and epithelial cells. On the other hand, Nagi et al. [26] revealed that HPV infection was present in healthy tissue, but it was more common in the malignant samples. All healthy tissue samples in this publication were EBV-negative. They found no association between the presence of HPV and/or EBV and anatomical locations, tumor grade, tumor stage, and lymph node involvement. Ebrahimian Shiadeh et al. [27] determined that there was no difference in the prevalence of HPV infection or EBV/HPV coinfection, and that the prevalence of EBV infection was significantly higher in malignant than in healthy tissue samples. They also did not find an association between EBV and/or HPV infection with gender, age, tissue class (fresh or formalin-fixed paraffin-embedded), microscopic features (well, moderately, poorly, or unknown differentiation), and sampling method (biopsy or surgery), as well. Fernandes et al. [28] found no significant association between the presence of EBV and/or HPV and clinicopathological characteristics (i.e., tumor stage, grade, anatomic location of the tumor, the number of positive lymph nodes, and the presence of metastasis) of the CRC cohort, but coinfection with EBV and more than two subtypes of HPV was established to be a predictor of advanced colorectal cancer stage. No conclusions could be drawn based on such partial, contradictory, and insufficiently convincing results. On the other hand, the potential role of coinfection must not be ruled out. Additionally, it is critical for future studies to include a comparison of the viral presence between healthy and tumor tissue from the same person, as a control. Although this could be difficult to carry out, it was available in the paper published by Salyakina and Tsinoremas [25].

Despite the above-described association between EBV or HPV infection and CRC evolution, and that available literature data point to an interplay between those two viruses in a possibly synergistic fashion, there is still insufficient epidemiological evidence to demonstrate this beyond doubt. If future results provide that evidence, it would benefit diagnostic and prophylactic procedures, and clinical practices, including screening for EBV and HPV in mucosa biopsies. Finally, EBV and HPV coinfections were suggested as “oncogenic collaborators” in oral, breast, tonsillar, and prostate cancer, which increases the importance of this scientific hypothesis [6].

The major limitation of this systematic review is the small number of available original publications and the quality of those included. Thus, an adequate and previously calculated sample size according to the main aim of the research with a multi-center design at the national level will be precious. On the other side, the possible influence of poor study quality could under- or over- estimate the overall effect in the meta-analysis. Based on the data provided by the included studies, it was possible to calculate the prevalence, but not the association between coinfection and CRC, the risk of CRC developing in patients with coinfection, or CRC clinicopathological characteristics and symptoms. Therefore, two important questions still await answers: whether coinfection is more common in CRC compared to healthy tissue, and whether EBV/HPV coinfection is associated with advanced tumor grade or stage, worse prognosis, or some other phenotypic characteristics of the cancer.

## 5. Conclusions

Although there are many advances in the diagnostic and therapeutic management of CRC, its poor prognosis still indicates the necessity of a better and deeper understanding of the pathogenesis. Increased knowledge about the infectious origin of various cancers, including colorectal cancer, has the potential to enhance the vigilance of scientists to design future research as large-scale multicenter case-control studies. This systematic review showed that there is not enough data and that they are heterogeneous, but it also showed a noteworthy prevalence of EBV/HPV coinfection in CRC and the undeniable need for further investigations of both its prevalence and role in oncogenesis, disease progression and prognosis.

## Figures and Tables

**Table 1:** Quality assessment.

Study	Selection Domain	Comparability Domain	Outcome/Exposure Domain	Quality
Salyakina, 2013 [25]	☆☆☆☆	☆	☆☆☆	Good
Gupta, 2020 [18]	☆		☆	Poor
Malki, 2020 [17]	☆		☆	Poor
Nagi, 2021 [26]	☆☆		☆	Poor
Ebrahimian-Shiadeh, 2023 [27]	☆☆☆☆	☆	☆☆	Good
Fernandes, 2023 [28]	☆☆		☆☆	Poor

**Table 2:** Characteristics of included studies.

Study Characteristics	CRC Patients’ Characteristics			Coinfected Patients’ Characteristics			EBV		HPV		
Author, Year Country Study Design	No	Age Gender (M/F, n (%))	Lymph Node Involvement Distant Metastasis	No	Histological Type Grade Stage	Localization	Tissue type Detection Method	No EBV+	HPV Genotype in Coinfections	Detection Method	No HPV+
Salyakina, 2013 [25] USA NR	165	NR NR	NR NR	13/165	Adenocarcinoma NR NR	Colon	Frozen tissue NGS	Ascending colon, n = 7 Descending colon, n = 0 Transverse colon, n = 1 Sigmoid colon, n = 19 Rectosigmoid, n = 0 Cecum, n = 13 Rectum, n = 22 Splenic flexure, n = 0 Hepatic flexure, n = 2	18	Frozen tissue NGS	Ascending colon, n = 11 Descending colon, n = 1 Transverse colon, n = 4 Sigmoid colon, n = 21 Rectosigmoid, n = 0 Cecum, n = 18 Rectum, n = 3 Splenic flexure, n = 1 Hepatic flexure, n = 3
Gupta, 2020 [18] Bosnia and Herzegovina NR	106	65 ± 8 (mean ± sd) 64 (60%)/42 (40%)	NR NR	17/106	Adenocarcinoma Intermediate and high grade Not clear	Rectum	FFPE PCR	26/106	16, 18, 31, 35, 45, 51, 52, 56, and 58	FFPE PCR	98/106 HPV16 53% HPV31 51% HPV18 50% HPV51 46% HPV52 39% HPV45 39% HPV35 26% HPV56 9% HPV39 0.9%
Malki, 2020 [17] Syria NR	102	49 (med) 49 (48%)/53 (52%)	NR NR	17/102	Adenocarcinoma Intermediate grade, n = 7; high grade, n = 10 NR	Not specified	FFPE PCR, TMA, IHC	20/102	16, 18, 31, 33, and 35	FFPE PCR, TMA, IHC	38/102
Nagi, 2021 [26] Lebanon NR	94	60 (3–89) mean (range) 37 (35%)/70 (65%)	Yes (pN0 n = 52, pN1 n = 20, pN2 n = 22, pN3 n = 0) NR	26/94	Adenocarcinoma Not clear Not clear	Rectosigmoid, n = 12 Other parts of the colon, n = 14	FFPE PCR, TMA, IHC	60/94	18, 33, 35, 52, and 58	FFPE PCR, TMA, IHC	27/94 HPV16 39% HPV18 38% HPV35 29% HPV58 28% HPV51 26% HPV45 23% HPV52 21% HPV31 14% HPV33 4%
Ebrahimian Shiadeh, 2023 [27] Iran cross-sectional	55	61 ± 12.8 mean ± sd 32 (58%)/23 (42%)	NR NR	1/55	Adenocarcinoma Not clear NR	Not specified	Frozen tissue PCR	27/55	NR	Frozen tissue PCR	4/55
Fernandes, 2023 [28] Qatar NR	100	57.1 ± 13.9 mean ± sd 23–96 range 66 (66%)/34 (34%)	Yes (pN0 n = 5, pN1 n = 7, pN2 n = 5) No	17/100 n = 7 EBV/One HPV, n = 4 EBV/Two HPVs, n = 1 EBV/Three HPVs, n = 3 EBV/Four HPVs, n = 2 EBV/Five HPVs	Adenocarcinoma Low grade, n = 0; intermediate grade, n = 12; high grade, n = 5 pT1 stage, n = 1 pT2 stage, n = 6 pT3 stage, n = 10 pT4 stage, n = 0	Ascending colon, n = 2 Descending colon, n = 1 Transverse colon, n = 1 Sigmoid colon, n = 5 Rectosigmoid, n = 1 Cecum, n = 1 Rectum, n = 2 Splenic flexure, n = 0 Hepatic flexure, n = 2	FFPE PCR	21/100 Ascending colon, n = 8 Descending colon, n = 7 Transverse colon, n = 3 Sigmoid colon, n = 18 Rectosigmoid colon, n = 9 Cecum, n = 4 Rectum, n = 2 Hepatic flexure, n = 5 Splenic flexure, n = 2 Other colorectal regions, n = 3	16, 18, 31, 35, 45, 51, 52, and 59	FFPE PCR	69/100 Ascending colon, n = 11 Descending colon, n = 10 Transverse colon, n = 3 Sigmoid colon, n = 18 Rectosigmoid colon, n = 10 Cecum, n = 6 Rectum, n = 2 Hepatic flexure, n = 4 Splenic flexure, n = 2 Other colorectal regions, n = 3

Abbreviations: M/F—Male/Female, CRC—colorectal carcinoma, EBV—Epstein–Barr virus, HPV—Human papillomavirus, PCR—Polymerase chain reaction, TMA—Tissue microarray, IHC—Immunohistochemistry, NGS–Next generation sequencing, FFPE—Formalin-fixed paraffin-embedded, NR—Not reported.

## Data Availability

The data presented in this study are available on request from the corresponding author.

## Supplementary Materials

The following supporting information can be downloaded at: https://www.mdpi.com/article/10.3390/microorganisms12112117/s1, Figure S1: Funnel plot. Figure S2: Sensitivity analysis with studies of poor quality [17,18,26,28]. Figure S3: Sensitivity analysis with studies performed in Asia [17,26,27,28]. Figure S4: Sensitivity analysis with studies using FFPE and PCR [17,18,26,27,28].
