# Supplementary material for: Epstein-Barr Virus and Human Papillomavirus Coinfection in Colorectal Carcinoma: Systematic Review and Meta-Analysis of the Prevalence
Source: Microorganisms. 2024 Oct 23;12(11):2117. doi: 10.3390/microorganisms12112117 (PMC11596815; doi:10.3390/microorganisms12112117)
Supplement: Supplementary file 1 [file microorganisms-12-02117-s001.zip › microorganisms-3225674-supplementary.pdf]

Supplement material

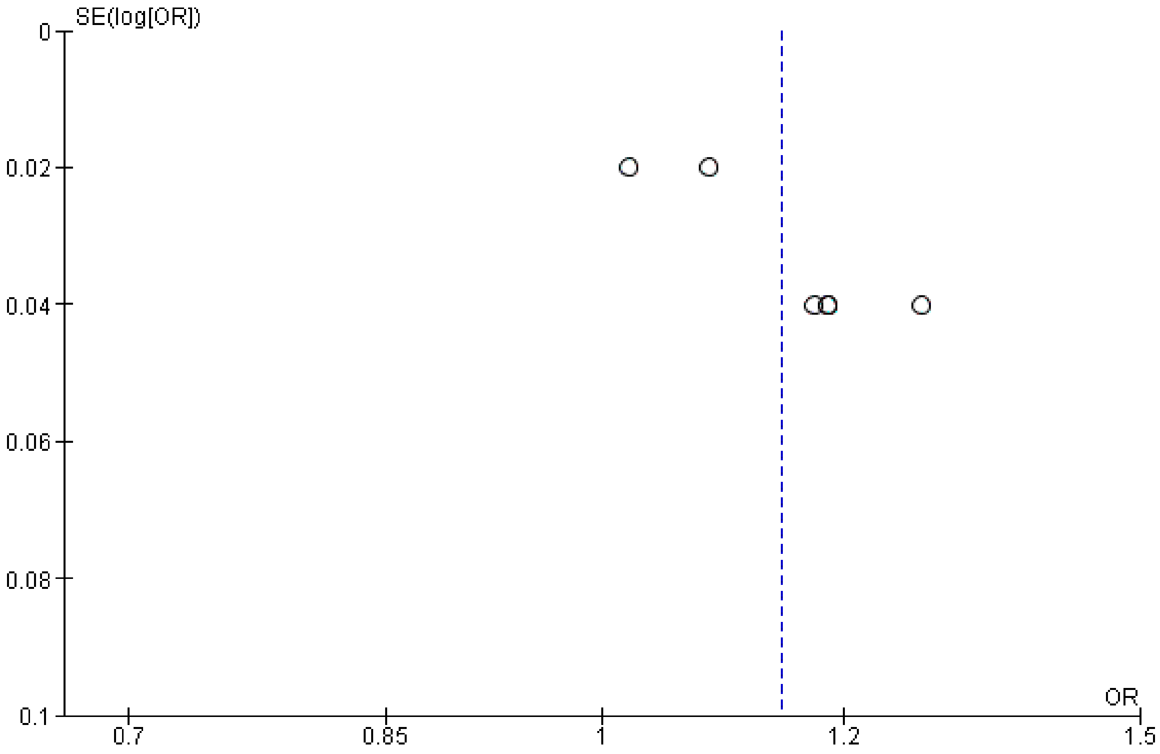

Figure S1. Funnel plot.

Study or Subgroup	log[Odds Ratio]	SE	Weight	Odds Ratio IV, Random, 95% CI	Year
Gupta, 2020	0.16	0.04	24.8%	1.17 [1.09, 1.27]	2020
Malki, 2020	0.17	0.04	24.8%	1.19 [1.10, 1.28]	2020
Nagi, 2021	0.28	0.05	23.3%	1.32 [1.20, 1.46]	2021
Fernandes, 2023	0.02	0.02	27.1%	1.02 [0.98, 1.06]	2023
**Total (95% CI)**			**100.0%**	**1.16 [1.04, 1.31]**	

Heterogeneity: $Tau^2 = 0.01$; $Chi^2 = 33.75$, df = 3 ($P < 0.00001$); $I^2 = 91\%$
Test for overall effect: Z = 2.56 (P = 0.01)

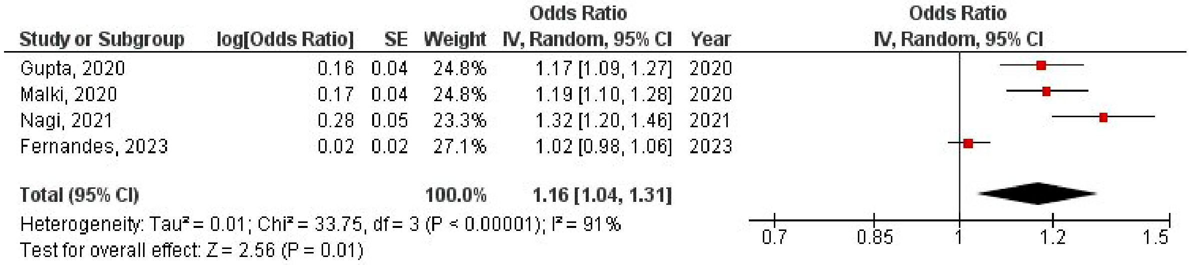

Figure S2: Sensitivity analysis with studies of poor quality [17,18,26,28].

Study or Subgroup	log[Odds Ratio]	SE	Weight	Odds Ratio IV, Random, 95% CI	Year
Malki, 2020	0.17	0.04	24.8%	1.19 [1.10, 1.28]	2020
Nagi, 2021	0.28	0.05	23.4%	1.32 [1.20, 1.46]	2021
Fernandes, 2023	0.02	0.02	27.0%	1.02 [0.98, 1.06]	2023
Ebrahimian Shiadeh, 2023	0.17	0.04	24.8%	1.19 [1.10, 1.28]	2023
**Total (95% CI)**			**100.0%**	**1.17 [1.04, 1.31]**	

Heterogeneity: $Tau^2 = 0.01$; $Chi^2 = 34.69$, df = 3 ($P < 0.00001$); $I^2 = 91\%$
Test for overall effect: Z = 2.57 (P = 0.01)

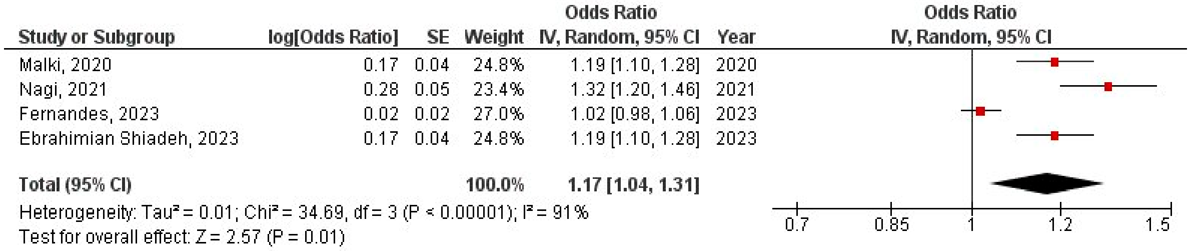

Figure S3: Sensitivity analysis with studies performed in Asia [17,26–28].

Study or Subgroup	log[Odds Ratio]	SE	Weight	Odds Ratio IV, Random, 95% CI	Year
Malki, 2020	0.17	0.04	19.8%	1.19 [1.10, 1.28]	2020
Gupta, 2020	0.16	0.04	19.8%	1.17 [1.09, 1.27]	2020
Nagi, 2021	0.28	0.05	18.5%	1.32 [1.20, 1.46]	2021
Fernandes, 2023	0.02	0.02	22.0%	1.02 [0.98, 1.06]	2023
Ebrahimian Shiadeh, 2023	0.17	0.04	19.8%	1.19 [1.10, 1.28]	2023
**Total (95% CI)**			**100.0%**	**1.17 [1.06, 1.29]**	

Heterogeneity: $Tau^2 = 0.01$; $Chi^2 = 37.33$, df = 4 ($P < 0.00001$); $I^2 = 89\%$
Test for overall effect: Z = 3.17 (P = 0.001)

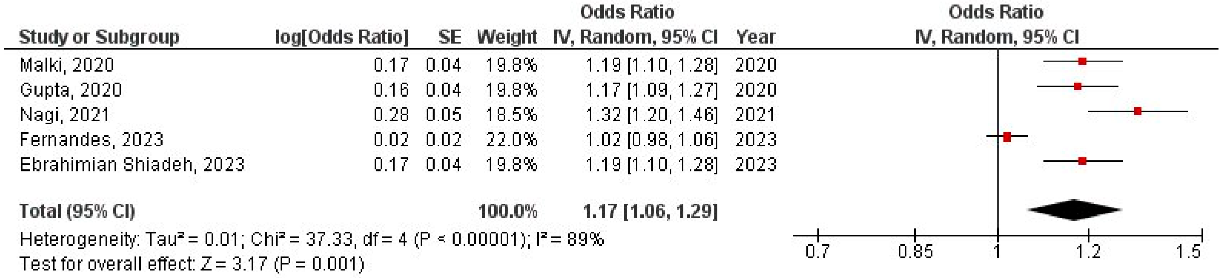

Figure S4: Sensitivity analysis with studies using FFPE and PCR [17,18,26–28].
